# Improving the trustworthiness, usefulness, and ethics of biomedical research through an innovative and comprehensive institutional initiative

**DOI:** 10.1371/journal.pbio.3000576

**Published:** 2020-02-11

**Authors:** Daniel Strech, Tracey Weissgerber, Ulrich Dirnagl, on behalf of QUEST Group

**Affiliations:** 1 QUEST Center for Transforming Biomedical Research, Berlin Institute of Health (BIH), Berlin, Germany; 2 Charité - Universitätsmedizin Berlin, Berlin, Germany

## Abstract

The reproducibility crisis triggered worldwide initiatives to improve rigor, reproducibility, and transparency in biomedical research. There are many examples of scientists, journals, and funding agencies adopting responsible research practices. The QUEST (Quality-Ethics-Open Science-Translation) Center offers a unique opportunity to examine the role of institutions. The Berlin Institute of Health founded QUEST to increase the likelihood that research conducted at this large academic medical center would be trustworthy, useful for scientists and society, and ethical. QUEST researchers perform “science of science” studies to understand problems with standard practices and develop targeted solutions. The staff work with institutional leadership and local scientists to incentivize and support responsible practices in research, funding, and hiring. Some activities described in this paper focus on the institution, whereas others may benefit the national and international scientific community. Our experience, approaches, and recommendations will be informative for faculty leadership, administrators, and researchers interested in improving scientific practice.

## Abstract 2

This Community Page article describes an ongoing large-scale structured initiative at the Berlin Institute of Health which aims at improving the robustness, reproducibility and transparency of research in an academic biomedical setting.

## Reducing waste and improving value in biomedicine: A role for institutions

Concerns about robustness, reproducibility, and transparency have prompted worldwide initiatives to reduce waste and increase value in biomedical research [1]. Important triggers for this movement included the high failure rates of the pharmaceutical industry when trying to replicate pivotal findings of academic researchers [2]. Potential “breakthrough” therapies, which are spectacularly successful in animal models of disease, very often failed in clinical trials. At the same time, meta-research has exposed substantial weaknesses in planning, conducting, analyzing, and reporting of biomedical research [3]. Scientists, clinicians, funders, journals, academies, regulators, and professional societies need to collaborate to make biomedical research more trustworthy and useful. Institutions play a pivotal role in these activities [4], yet there is little information available on what a comprehensive institutional intervention might look like or whether institutional interventions are effective.

In 2017, the Berlin Institute of Health (BIH) founded the QUEST (Quality-Ethics-Open Science-Translation) Center to improve the quality and ethics of research conducted at the institution. Box 1 provides an overview of the center. In this paper, we share our experiences in implementing this large-scale, structured initiative designed to address concerns related to the reproducibility crisis at a large academic medical center, combined with a basic biomedical research institute. We believe that despite local and national idiosyncrasies, our experience and recommendations will be informative for researchers, faculty leadership, administrators, and others.

**Box 1. A brief overview of QUEST**

- Mission: In 2017, the BIH founded the QUEST Center to improve the trustworthiness, usefulness, and ethics of BIH research.
- Institution: QUEST activities focus on the BIH, which brings together 2 large biomedical research organizations. Charité–Universitätsmedizin Berlin is Europe's largest university hospital, with more than 4,000 scientists/doctors and 7,500 students. The Max Delbrück Center for Molecular Medicine (MDC) is a leading basic research institution that focuses on molecular biology and genetics. More than 300 postdocs and 300 PhD students work at the MDC. Researchers at these 2 institutions perform basic, translational, and clinical research.
- Structure: QUEST is divided into 2 parts, the office staff and the research team. These 2 groups often work collaboratively. QUEST also has an international advisory panel and hosts several international visiting fellows.
- QUEST Office: The office conducts and evaluates interventions at the BIH; however, some activities also have national or international impact. The office includes administrative staff and consultants on topics such as incentives and indicators, open data, education, and data science (8 full-time equivalent positions).
- QUEST Research: The research team aims to develop evidence, policies, and tools for increasing the value of biomedical research locally, nationally, and internationally (presently 17 full-time equivalent positions).
- Funding: The BIH provides institutional funding for QUEST. Individual projects are funded by public research funders.
- Affiliated Centers: Since 2019, QUEST has hosted the “Meta-Research Innovation Center Berlin” (METRIC Berlin; Director John P. A. Ioannidis, Stanford University). METRIC Berlin is funded by the Stiftung Charité and the Einstein Foundation Berlin. QUEST also hosts Collaborative Approach to Meta-Analysis and Review of Animal Data from Experimental Studies (CAMARADES) Berlin. CAMARADES provides support for groups conducting systematic reviews and meta-analyses of data from experimental animal studies.
- Outcomes: QUEST activities are designed to reduce waste and increase value of research at our institution. As the center has only been open for 3 years, we cannot provide evidence of efficacy as measured by endpoints such as “improved reproducibility,” “less translational attrition,” or “greater benefit to patients.” In this paper, we list proxies to demonstrate the impact of our efforts.

## The QUEST center: From theory to action

**Fig 1:**
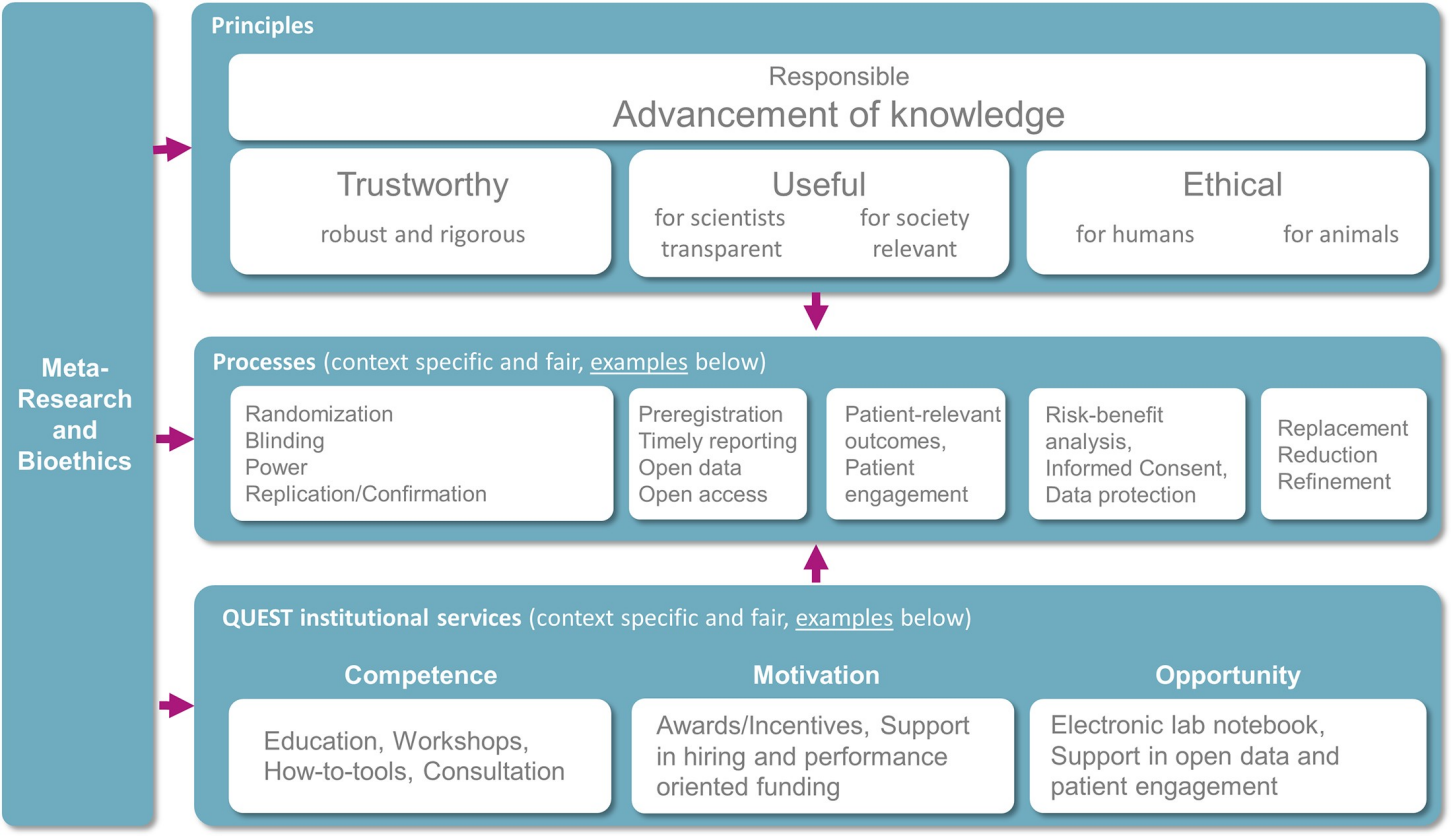
The QUEST Center framework for institutional “culture change”. The figure presents the principles and processes underpinning the rationale for all QUEST activities in line with the European Union principles of RRI. The processes outlined in the figure are examples and are not intended to be comprehensive. RRI, Responsible Research and Innovation; QUEST, Quality-Ethics-Open Science-Translation.

The scientific process is designed to advance knowledge; however, the reproducibility crisis began with scientists raising concerns that many published research findings are false [5]. The QUEST Center seeks to address this problem by encouraging researchers to adopt processes to increase the likelihood that research will be trustworthy, useful for scientists and society, and ethical for humans and animals (Fig 1). For example, researchers can improve the trustworthiness of research by using methods to reduce the risk of bias, such as randomization, blinding, power calculations, and replication studies. Strategies to make research more transparent and useful to scientists include preregistration, timely reporting of clinical trial results, open data, and open access. Experts, funders, and organizations have advocated for researchers to adopt the processes outlined in Fig 1, along with many others [6–8], yet these practices are not widely accepted in many fields. Institutions can play a role in shifting established mindsets and behaviors by offering innovative services to incentivize, support, and evaluate the uptake of responsible research practices.

## Understanding the problem: Research on research

Research on research (or meta-research) studies provide essential information that helps us to set priorities for the QUEST Center and determine whether interventions are working. These studies allow us to understand and quantify problems with standard practices at our institution, as well as on a national and international level. We then use this information to develop targeted solutions. For example, we obtained baseline data on research culture by conducting a validated survey of approximately 7,000 researchers and clinicians at our institution [9]. We asked clinicians and researchers how available advisors or supervisors are to their advisees/supervisees, or how true it is that pressure to publish has a negative effect on the integrity of research in their research environment. We will repeat this survey in a few years to assess changes. On a national level, QUEST researchers recently examined rates of timely reporting for clinical trial results among all German universities. At most institutions, only 40% of clinical trial results are available within 2 years of trial completion [10]. On the basis of these data, we held a workshop to train clinicians and administrators from German universities to implement procedures to improve timely reporting of clinical trials at their institutions. QUEST researchers have also used meta-research studies to quantify problems with published papers in fields such as preclinical stroke research, physiology, or peripheral vascular disease. These educational papers highlight solutions to common problems, such as failing to report the number of excluded animals and reasons for exclusion [11], using misleading bar graphs to present continuous data [12], and inadequate reporting of statistical methods [13]. Studies by QUEST researchers have been valuable in changing standards for the scientific community. For example, an increasing number of journals and publishers are implementing policies that encourage or require authors to replace bar graphs of continuous data with more informative graphics (i.e., dot plots, box plots, histograms) [14]. Although we can only highlight a few research projects here, our website (https://quest.bihealth.org) contains further information on meta-research and translational bioethics research at QUEST. An online document provides a detailed overview of QUEST activities (https://osf.io/kqr5y/), illustrating the relationship between meta-research and QUEST services that may benefit the institutional, national, and international research communities.

## Changing science at the institutional level

**Fig 2:**
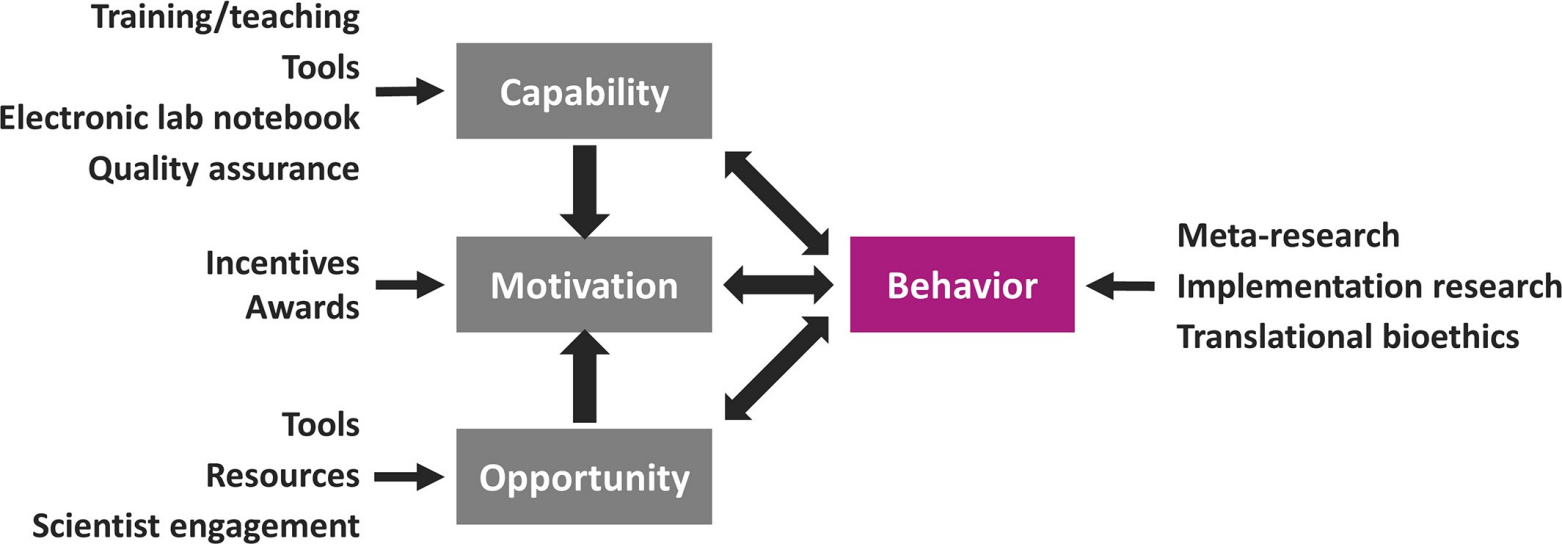
Using QUEST services to facilitate behavior change. The figure illustrates how the approaches used by the QUEST Center (in black) map onto a simple model of behavior change (modified after Michie et al., Implementation Science 2011, 6:42). QUEST, Quality-Ethics-Open Science-Translation.

The QUEST services described next highlight the scope of QUEST activities and provide examples of active engagement with the local research community. In order to implement new ideas, we need to specifically adapt them to the idiosyncrasies of the Berlin institutional research environment and local governance structures. Conceptually, we are conducting and evaluating a large-scale behavior change intervention. Fig 2 illustrates how QUEST activities map onto an established model of behavior change.

### Training, teaching, and outreach

We develop training and teaching resources and offer courses on experimental and study design, on methods to reduce bias, and on new modes of publishing. Importantly, these courses are offered to researchers at all academic career stages. Furthermore, we provide an expanding portfolio of local, national, and international workshops and seminars on topics such as “Reproducible science with R,” “Good practice in Peer Review,” and “Best practice in preclinical animal research.” Recordings of some seminars are publicly available through our media center (https://www.bihealth.org/de/forschung/quest-center/mediathek/). Early career researchers from around the world can apply to attend the Berlin-Oxford Summer School, a yearly workshop focused on reproducible research practices.

We are testing new approaches to change practices of individual researchers and research groups. We give short introductory talks to departments and lab groups that highlight responsible research practices, as well as QUEST services and resources. We are also developing a “spoke and hub” model to train researchers to adopt responsible practices and share their skills with their colleagues.

### Accessibility and transparency

The QUEST Center aims to increase the accessibility and transparency of research at our institution by promoting open science and high-quality research data management. Besides working to popularize open access, we promote open data through a combination of awards, tools, workshops, and the development of an institutional research data policy focused on research data management. We further created an automated screening tool that uses text mining to identify papers containing open data (ODDPub, [15]). Investigators who publish open data automatically receive a financial reward.

### Quality assurance

We promote quality assurance in preclinical research with regard to standards on the design, conduct, analysis, and reporting of experiments. We are developing a biomedically oriented quality management system that is open source, modular, and simple and appeals to academic researchers (PREMIER, [16]).

### Incentives and indicators

In consultation with Charité leadership, we use incentives to raise awareness and nudge clinicians and researchers toward adopting responsible research and open science practices. For example, any institutional investigator can apply to receive a 1,000-Euro research bonus for publishing a null result, a replication study, a preregistered preclinical study, a paper that reuses data previously published by others, or a study that included patient engagement.

The QUEST office also works with the BIH and Charité leadership to consider responsible research practices when evaluating applications for funding, hiring, and tenure. Since 2018, Charité professorship applicants have been required to answer additional questions related to responsible research practices, including publication of null results, open data, and stakeholder engagement. QUEST office staff screen applications and participate in hiring committee meetings to support committee members in understanding, evaluating, and applying the new criteria. These criteria are also used to evaluate applications for intramural funding schemes.

### Tools and resources

We also provide tools and resources for robust and useful research. These tools include an electronic laboratory notebook [17] and a Laboratory Critical Incidence Reporting System (LabCIRS, [18]). Our toolbox also includes File-Drawer Data Liberation Effort (FIDDLE), a Shiny app that provides guidance on where and how to publish null, inconclusive, negative, and other “nonstandard” results. With the exception of the electronic lab notebook, all of these resources are open source and publicly accessible.

## Sharing resources and experiences with the global scientific community

Throughout the paper, we have highlighted several ways in which QUEST research, tools, and resources may benefit the global scientific community. Table 1 highlights resources that may be particularly valuable to those interested in using the tools discussed here or in implementing some of these interventions at their own institutions. Box 2 summarizes important lessons that we have learned over the past 3 years, which may be useful to those at other institutions who have similar goals.

**Box 2. Lessons learned during the first 3 years of QUEST**

- In Germany, all departments follow systems and procedures established by a central administration. **Strong support from institutional leaders** is essential to change reward and incentive structures while emphasizing that responsible research is an essential part of the institutional mission.
- **Highlight official recommendations** from national or international funders, journals, and guidelines to increase awareness, credibility, and compliance.
- **Provide “benchmarking” data on institutional or national performance** on responsible research metrics, which raises awareness of problems and encourages the research community to implement existing solutions or develop new ones. Examples of benchmarking include demonstrating compliance with international guidelines for timely reporting or open access and internal validity indicators of published studies (such as use of randomization, blinding).
- **Create a positive narrative by emphasizing improving value** over reducing waste while **offering concrete advice or help**. Communication should never be accusatory, and interventions should be supportive, rather than punitive.
- **Top-down approaches need to be combined with bottom-up engagement** of researchers, clinicians, technicians, administrators, and students to build community support. For example, the QUEST SPOKES program stimulates grassroots activities by engaging motivated early and midcareer researchers and enabling them to serve as ambassadors who promote a value-oriented research culture.
- Collaborate with local research groups and regional, national, or international initiatives to **address ongoing challenges and increase dissemination**.
- Almost everyone in the academic system is willing to do “the right thing,” but most are overcommitted and lack resources and knowledge or expertise. Assistance via **services and other forms of support** (e.g., protected time) is essential to facilitate change.

**Table 1:** Examples of tools and resources of interest to the international research community (a more comprehensive and updated list is given at https://osf.io/kqr5y/).

Resource Name	Details
QUEST Toolbox	Find tools, programs, and online platforms for conducting reproducible research at all stages of a research project http://bit.ly/Quest-Toolbox.
FIDDLE	This tool is designed to help researchers to get data out of the file drawer and into the scientific literature. Find out where and how to publish data from well-designed experiments that are difficult to publish in traditional journals (i.e., null results, inconclusive results, datasets, etc. [19]).
Digital open science-teaching tool for reproducible and transparent research	An introductory course that guides students toward a reproducible science workflow. Outline of course content and possible extensions, including encountered challenges and a discussion on how to integrate such a course in existing curricula [20].
ODDPub	We use this tool to automatically screen for open data in all papers by researchers from our institution. We then incentivize data sharing by issuing small monetary rewards to investigators who published papers with open data [15].
QUEST Criteria	Additional criteria for the assessment of research. These responsible research criteria are used to evaluate professorship candidates and intramural funding applications. We are continuing to develop these criteria based on our experiences with hiring commissions and intramural funding schemes [21].
GOT-IT	GOT-IT provides a fit-for-purpose, flexible set of guidelines on robust drug target validation. These guidelines are suitable for implementation in an academic setting and include an education program as well as an online expert platform (https://got-it.app/).
LabCIRS	LabCIRS is a simple, free, open-source software tool for implementing a critical incidence and error reporting system in research groups, laboratories, or large institutions [18].

Abbreviations: FIDDLE, File-Drawer Data Liberation Effort; GOT-IT, Guidelines on Target Validation for Innovative Therapeutics; LabCIRS, Laboratory Critical Incident and Error Reporting System; ODDPub, Algorithm for detecting open data in scientific publications; QUEST, Quality-Ethics-Open Science-Translation

We believe that institutions have an important role in changing biomedical research; however, it’s important to recognize that there are limitations to what a single institution can achieve. Although our activities may affect the local research environment, changing national and international evaluation standards is a more complex and time-consuming process. In the absence of widespread, coordinated changes, what might be good for a career at our institution may be neutral or even negative when moving to another institution. The success of the global scientific community in increasing the value of biomedical research thus strongly depends on a large-scale alliance of research institutions and organizations that have made long-term commitments to changing scientific culture and practice. To this end, the QUEST Center is networking with other universities (e.g., University of Oxford, United Kingdom; European University Hospital Alliance), funders (e.g., Wellcome Trust, UK), and other stakeholders (e.g., Reduce Research Waste And Reward Diligence [REWARD] and Enhancing the Quality and Transparency of Health Research [EQUATOR] Networks; UK Reproducibility Network) both nationally and internationally.

## Conclusions

The QUEST Center was established to support emerging efforts to increase value and reduce waste in biomedical research. The QUEST office is translating these efforts at one of the largest biomedical treatment, education, and research centers in Europe. In addition to developing a framework for our activities (Fig 1), we have worked to improve research practices and change the scientific culture by implementing measures derived from this framework. Although it is still too early for a meaningful assessment of the overall outcomes and impact of QUEST activities, future publications will present results from ongoing program evaluations. We have already demonstrated, however, that it is feasible to devise, implement, and evaluate interventions designed to improve the quality and value of translational research in a large academic medical center.

## Supporting information

S1 Authors List of QUEST Group authors. (DOCX)

## Abbreviations

BIH: Berlin Institute of Health
CAMARADES: Collaborative Approach to Meta-Analysis and Review of Animal Data from Experimental Studies
FIDDLE: File-Drawer Data Liberation Effort
LabCIRS: Laboratory Critical Incidence Reporting System
MDC: Max Delbrück Center for Molecular Medicine
METRIC Berlin: Meta-Research Innovation Center Berlin
RRI: Responsible Research and Innovation
QUEST: Quality-Ethics-Open Science-Translation
