# Supplementary material for: Improving the trustworthiness, usefulness, and ethics of biomedical research through an innovative and comprehensive institutional initiative
Source: PLoS Biol. 2020 Feb 11;18(2):e3000576. doi: 10.1371/journal.pbio.3000576 (PMC7012388; doi:10.1371/journal.pbio.3000576)
Supplement: S1 Authors — (DOCX) [file pbio.3000576.s001.docx]

QUEST Group:

Rene Bernard^1,2^, Merlin Bittlinger^1,2^, Evgeny Bobrov^1^, Meggie Danziger^1,2^, Ulrich Dirnagl^1,2^*****, Klaus-Detlef Heber^1^, Peter Grabitz^1,2^, Miriam Kip^1^, Corinna Klingler^1,2^, Holger Langhof^1,2^, Sarah K McCann^1,2^, Stephanie Müller-Ohlraun^1^, Elena Pavlenko^1,2^, Ingo Przesdzing^1,2^, Nico Riedel^1^, Bob Siegerink^1,2^, Daniel Strech^1,2^, Ulf Toelch^1^, Tracey Weissgerber^1,2^, Sarah Weschke^1^, Christiane Wetzel^1,2^

1 QUEST Center for Transforming Biomedical Research, Berlin Institute of Health (BIH), Berlin, Germany

2 Charité - Universitätsmedizin Berlin, Berlin, Germany
